# Mass Azithromycin and Malaria Parasitemia in Niger: Results from a Community-Randomized Trial

**DOI:** 10.4269/ajtmh.16-0487

**Published:** 2017-06-19

**Authors:** Kieran S. O'Brien, Sun Y. Cotter, Abdou Amza, Boubacar Kadri, Baido Nassirou, Nicole E. Stoller, Zhaoxia Zhou, Chris Cotter, Sheila K. West, Robin L. Bailey, Philip J. Rosenthal, Bruce D. Gaynor, Travis C. Porco, Thomas M. Lietman

**Affiliations:** 1 Francis I. Proctor Foundation, University of California San Francisco, San Francisco, California;; 2 Programme FSS/Université Abdou Moumouni de Niamey, Programme National de Santé Oculaire, Niamey, Niger;; 3 Global Health Group, University of California San Francisco, San Francisco, California;; 4 Dana Center for Preventive Ophthalmology, Wilmer Eye Institute, Johns Hopkins University, Baltimore, Maryland;; 5 Clinical Research Unit, Department of Infectious and Tropical Diseases, London School of Hygiene and Tropical Medicine, London, United Kingdom;; 6 Department of Medicine, University of California San Francisco, California;; 7 Department of Ophthalmology, University of California San Francisco, San Francisco, California;; 8 Department of Epidemiology and Biostatistics, University of California San Francisco, San Francisco, California

## Abstract

Studies designed to determine the effects of mass administration of azithromycin on trachoma have suggested that mass azithromycin distributions may also reduce the prevalence of malaria. These studies have typically examined the impact of a small number of treatments over short durations. In this prespecified substudy of a cluster-randomized trial for trachoma, we compared malaria parasitemia prevalence in 24 communities in Niger randomized to receive either annual or biannual mass azithromycin distributions over 3 years. The 12 communities randomized to annual azithromycin received three treatments during the high-transmission season, and the 12 communities randomized to biannual azithromycin received a total of six treatments: three during the high-transmission season and three during the low-transmission season. Blood samples were taken to assess malariometric indices among children in all study communities at a single time point during the high-transmission season after 3 years of the intervention. No significant differences were identified in malaria parasitemia, parasite density, or hemoglobin concentration between the annual and biannual treatment arms. When compared with annual mass azithromycin alone, additional mass azithromycin distributions given during the low-transmission season did not significantly reduce the subsequent prevalence of malaria parasitemia or parasite density after 3 years, as measured during the high-transmission season.

## INTRODUCTION

Azithromycin has been proposed as a potential alternative or adjunctive option for use in the prevention and treatment of uncomplicated malaria.^1^^–^^6^ Though azithromycin does have antimalarial properties, evidence suggests that its activity against malaria is weak and inferior to first-line agents as monotherapy, and support for its use in combination with other antimalarial drugs remains equivocal.^7^^,^^8^ Azithromycin is well tolerated, however, and is considered to have minimal risk associated with use in young children and pregnant women.^8^^–^^10^

Mass distributions of oral azithromycin are currently used to control ocular strains of *Chlamydia* in trachoma-endemic areas. Studies have also identified potential secondary benefits of mass azithromycin given for trachoma among children, including reductions in the prevalence of malaria and its sequelae.^11^^–^^14^ These prior trachoma studies examined the short-term effects of a small number of mass distributions of azithromycin on malaria. Trachoma programs typically give multiple rounds of azithromycin over the course of several years, however, and the longer term effects of these mass distributions on malaria are unclear.

A previously published substudy of a large, cluster-randomized trial for trachoma conducted in Niger found that malaria parasitemia among children was significantly lower in communities that received two mass distributions of azithromycin compared with communities that received one distribution.^11^^,^^15^ This study suggested that an additional mass distribution during the low-transmission season contributed to the lower prevalence found after 1 year of treatment.^11^ Herein, we assess malaria parasitemia among children in Niger after 3 years of treatment in a different set of communities randomized to annual or biannual mass distributions of oral azithromycin. This time frame enabled us to examine longer term effects of multiple distributions of azithromycin on malaria when given during both the low- and high-transmission seasons.

## MATERIALS AND METHODS

### Study setting and participants.

The Partnership for the Rapid Elimination of Trachoma was a group of cluster-randomized trials conducted in The Gambia, Niger, and Tanzania (clinicaltrials.gov, NCT00792922).^15^ Eligibility criteria for study communities have been described in depth previously.^16^ Niger communities were eligible for inclusion if the total population was between 250 and 600 people at the time of the last government census and the prevalence of active trachoma among children under 72 months of age was ≥ 10%. In Niger, participants from 48 grappes (smallest government health unit, termed “community” in this report) in six Centers de Santé Intégrées (CSIs) in the Matameye District of the Zinder region were included in the trial.

### Trial design and randomization.

A 2 × 2 factorial design was used to evaluate both varying treatment frequencies and targeted treatment coverage levels. Communities were randomized into four arms with 12 communities each: 1) annual treatment at standard (80%) coverage, 2) annual treatment at enhanced (90%) coverage, 3) biannual treatment at standard (80%) coverage, and 4) biannual treatment at enhanced (90%) coverage. In this report, the two enhanced coverage arms comparing annual to biannual treatment at a target coverage of 90% are examined (2 and 4 in the preceding list). Within each CSI, stratified blocked randomization of communities was performed based on clinical trachoma prevalence in children as described previously.^16^ An annual census was conducted before each annual treatment in all study communities over 3 years. Participants were monitored for trachoma biannually. Malaria assessments were conducted in 24 communities from the two enhanced coverage arms after 3 years of treatment.

The statistical package R (version 2.12; R Foundation for Statistical Computing, Vienna, Austria; www.r-project.org) was used to generate the random allocation sequence of clusters (Travis C. Porco).^16^ Study staff used MS Access (version 2007; Microsoft Corp., Redmond, WA) to randomly select individuals for trachoma and malaria assessments.

### Intervention.

Among the 24 enhanced coverage communities discussed herein, 12 were randomized to receive annual mass distribution of oral azithromycin in all persons ≥ 6 months of age, and 12 were randomized to receive biannual mass distribution of oral azithromycin in children 6 months to 12 years of age during the 3-year study period. Annual distributions were provided during the high-transmission season for malaria in Niger, and the additional biannual distributions were provided during the low-transmission season. In both arms, treatment entailed directly observed doses of oral azithromycin (20 mg/kg up to a maximum dose of 1 g in adults).^17^^,^^18^ Children under 6 months of age and those allergic to macrolides were given topical tetracycline ointment (1%) to be applied to both eyes twice a day for 6 weeks. In the annual treatment arm, pregnant women were also offered topical tetracycline. Treatment coverage was determined based on the census directly preceding treatment.

Community residents were advised to alert village health workers within 2 weeks after mass treatment if they and/or their children experienced an adverse event, defined as diarrhea, nausea, and vomiting for more than 2 days, hospitalization for any cause, or death. Residents were referred to the nearest health-care center as needed.

### Clinical and laboratory assessments.

Blood samples were collected from children in both the annual and biannual treatment arms at 36 months after study initiation. The study aimed to collect thick blood smears and hemoglobin concentration from 50 children in each study community. Sixty-two children 6–60 months of age from each community were randomly selected from the most recent census prior to field data collection. If a community had fewer than 50 children, blood specimens were taken from all children. After obtaining verbal consent from a parent or guardian for each study participant, thick blood smears and hemoglobin concentration were collected at a centralized exam station in each community according to methods previously described.^11^

Thick blood smears were collected on glass slides, air-dried, and stored at room temperature. Two experienced microscopists at the Zinder Regional Hospital in Niger stained the thick blood smears with 3% Giemsa and used a light microscope to determine the presence or absence of *Plasmodium* parasites on the slides. The microscopists were masked to treatment arm. If both microscopists observed parasites, then the smear was considered positive. Discordant slides were considered negative. To assess parasite density, the microscopists counted the number of asexual parasites per 200 white blood cells (assuming white blood cell count = 8,000/μL).^19^ The average of the two parasite density readings was used in analyses. Gametocytes were considered present if observed by either microscopist, given the low prevalence. Hemoglobin concentration was determined for all randomly selected children (HemoCue AB, Ängelholm, Sweden).

### Sample size and statistical analysis.

We estimated that 24 communities (12 communities per arm) would provide greater than 80% power to detect a 3% absolute difference in malaria parasitemia. The sample size calculation assumed a baseline prevalence of 10% and an intraclass correlation coefficient (ICC) of 0.075.^20^

The primary analysis accounted for clustering at the level of the randomization unit (community) by comparing the community prevalence of malaria parasitemia in the annual and biannual treatments at 36 months after study initiation arms using a paired *t* test. To ensure results were not dependent on assumptions, sensitivity analyses were conducted with nonparametric tests (Wilcoxon signed rank) at the community level. A mixed-effects logistic regression model was used to examine effects at the individual level while clustering for community. Secondary outcomes, parasite density, gametocytemia, and hemoglobin concentration, were assessed similarly. All analyses were performed as intent-to-treat using Stata 13 (Statacorp LP, College Station, TX).

### Ethics statement.

Ethical approval was obtained from the University of California, San Francisco Committee for Human Research and the Comité d’Ethique du Niger (the Ethical Committee of Niger). This study is registered at clinicaltrials.gov (NCT00792922) and was implemented in accordance with the Declaration of Helsinki. Given the high rates of illiteracy in the study area, the institutional review boards approved verbal informed consent, both from the local chiefs of each community before randomization and each child participant’s parent or guardian before data collection.

## RESULTS

### Participants and treatment coverage.

**Figure 1.**
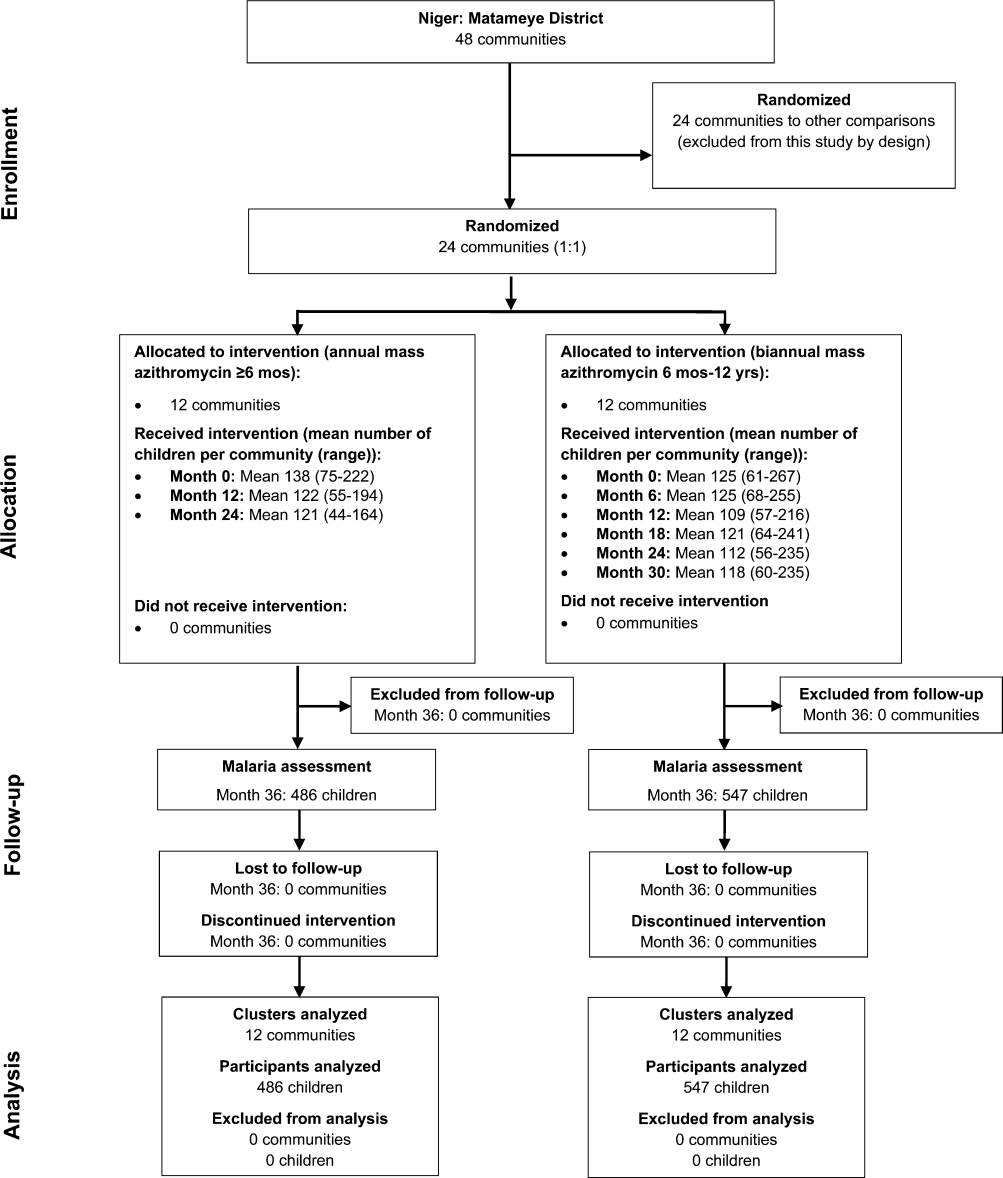
Participant flow in the Partnership for the Rapid Elimination of Trachoma cluster-randomized trial in Niger.

From May 2010 to September 2013, 24 communities in the enhanced (90%) coverage arm of the main Niger trial were followed. At baseline, the 12 communities randomized to annual mass azithromycin had a mean of 138 children (range, 75–222) and the 12 biannually treated communities had a mean of 125 children (range, 61–267) 6–60 months of age (Figure 1). As shown in Table 1, baseline characteristics of eligible children were comparable between treatment arms.

**Table 1:** Baseline characteristics of children ≤ 30 months at time of enrollment in 24 communities randomized to annual or biannual mass azithromycin in a cluster-randomized trial in Niger

Characteristic	Mean (95% confidence interval or range)	
	Annual azithromycin *N =* 12 communities	Biannual azithromycin *N =* 12 communities
Children per community	72 (range, 37–119)	66 (range, 36–124)
Proportion female (%)	52.1% (49.3–54.8)	49.0% (45.1–52.9)
Age (months)	18.4 (17.3–19.4)	18.7 (17.4–19.9)
Prevalence of TF*	23.4% (14.9–32.0)	17.6% (12.5–22.7)
Prevalence of TI*	7.1% (1.0–13.3)	5.0% (1.9–8.2)

* Trachomatous inflammation—follicular (TF) and trachomatous inflammation—intense (TI) assessed according to the World Health Organization Simplified Grading System.

All communities received three mass antibiotic distributions during the high-transmission season (June/July 2010, June/July 2011, and June/July 2012). The 12 communities randomized to biannual mass azithromycin were treated an additional three times during the low-transmission season (December 2010/January 2011, December 2011/January 2012, and December 2012/January 2013). Treatment coverage is shown in Table 2. The mean antibiotic coverage of children 6–60 months of age was greater than 90% across all treatment periods in both arms. All study communities were treated per protocol and no communities were lost to follow-up. No serious adverse events were reported during the study period.

**Table 2:** Average antibiotic treatment coverage in children 6–60 months of age in 24 communities in Niger over 3 years

Study arm	Mean (95% confidence interval)						
	0 months	6 months	12 months	18 months	24 months	30 months	36 months
Annual	95.5% (93.7–97.0%)	N/A	92.2% (88.9–94.5%)	N/A	92.0% (88.3–94.2%)	N/A	90.6% (86.5–93.5%)
Biannual	94.4% (92.3–96.0%)	92.1% (89.2–94.4%)	94.0% (92.3–95.5%)	92.6% (87.8–95.5%)	90.7% (89.0–92.2%)	91.0% (87.6–93.5%)	91.3% (88.3–93.8%)

### Assessments of malaria parasitemia and hemoglobin levels.

Children from each study community were randomly selected for this substudy from the most recent census in May 2013. Thick blood smears were collected from a total of 1,032 children in 24 communities (485 in the annual treatment arm; 547 in the biannual treatment arm) 3 years after enrollment (September 2013, high-transmission season). Hemoglobin concentration was collected from 1,033 children (486 in the annual treatment arm; 547 in the biannual treatment arm). Specimen collection occurred more than 1 year after the last treatment in the annually treated communities and approximately 8 months after the last treatment in the biannually treated communities.

Grades for the presence or absence of malaria parasites were concordant between the two microscopists in 99.4% of samples with an ICC of 0.994 (95% confidence interval [CI] = 0.993–0.995). Comparing the two assessments of parasite density resulted in an ICC of 0.957 (95% CI = 0.951–0.962). Table 3 shows the results for all assessments. At the single time point after 3 years of mass azithromycin distribution, the community-level prevalence of parasitemia was similar in the biannually treated communities and the annually treated communities (mean difference = 0.00, 95% CI = −0.15 to 0.15, *P* = 0.995). A mixed-effects logistic regression model with treatment arm as a fixed effect and community as a random effect showed similar results (odds ratio = 1.00, 95% CI = 0.58–1.73, *P =* 0.997). No significant differences were seen in community-level parasite density, gametocytemia, or hemoglobin concentration when comparing annually treated communities to biannually treated communities. Analyses conducted with Wilcoxon signed rank tests and mixed-effects regression models for each of these three assessments showed similar results.

**Table 3:** Results from blood assessments among children 6–60 months of age in 24 communities in Niger randomized to annual or biannual mass azithromycin over 3 years*

Measurement	Mean or % (95% confidence interval)		*P* value*
	Annual azithromycin *N =* 12 communities	Biannual azithromycin *N =* 12 communities	
Malaria parasitemia	54.5% (43.0–66.1%)	54.5% (44.8–64.2%)	0.995
Parasite density, parasites/μL^2^	7,710 (4,670–10,800)	4,930 (3,320–6,550)	0.11
Hemoglobin, g/dL	9.4 (9.1–9.6)	9.4 (9.1–9.7)	0.87
Gametocytemia	0.5% (0–1.3%)	0.7% (0–1.3%)	0.63

Blood assessments were conducted 36 months after enrollment.

* Paired *t* test.

† Parasite density measures rounded to the nearest ten.

## DISCUSSION

In this cluster-randomized trial, we were unable to demonstrate a difference in malaria parasitemia, parasite density, or hemoglobin concentration between children who received annual or biannual treatment with azithromycin after 3 years of the intervention. A previous study in Niger found a significant reduction in malaria parasitemia and parasite density in communities with an additional treatment during the low-transmission season compared with communities with a single mass treatment after 1 year.^11^ In this study, we assessed the effect of mass distributions of azithromycin on malaria in a different set of communities over a longer time period during the high-transmission season.

There are several possible explanations for the discrepancy in results between annual and biannual azithromycin after 1 year compared with 3 years. Azithromycin may lose effectiveness after multiple mass treatments due to increasing resistance. A previous study suggested that azithromcyin–artesunate treatment failure may have resulted from *Plasmodium* species resistance after mass azithromycin for trachoma in the area.^21^ Another study, however, failed to identify markers of resistance to azithromycin on a gene suspected to be involved in azithromycin resistance in vitro, though this study examined resistance after a single mass treatment.^13^ In general, little is known about parasite resistance to macrolides.

Another explanation for the difference in study results could be that the previous study assessed malaria outcomes in the low-transmission season and the present study assessed outcomes in the high-transmission season. Single mass treatments with azithromycin have shown reductions in malaria prevalence immediately after treatment, but these reductions are not maintained over time without additional treatment.^14^ An effect of azithromycin might be expected during the low-transmission season when reinfection is uncommon, but the lack of an effect during the high-transmission season may be due to the high malaria risk that remains even after azithromycin’s protective effects decline. Alternatively, the significant reduction in malaria parasitemia seen in the biannual treatment arm after 1 year may have been due to chance.

This study has several important limitations. First, no baseline assessment of the prevalence of malarial parasitemia was conducted, which may have revealed whether parasitemia decreased in both arms. Baseline prevalence could have offered a more powerful study design, though randomized posttest analysis does permit valid inference,^22^ since treatment assignments are stochastically independent of other explanatory covariates.^23^ In addition, the treatment schedule and dosage for the study was designed for trachoma control, not malaria prevention. Seasonal malaria chemoprevention has demonstrated efficacy when administered during the high-transmission season in areas like Niger with seasonal malaria.^24^^,^^25^ However, a mathematical model of malaria transmission dynamics in Niger demonstrated that mass azithromycin distributions during the high-transmission season may not necessarily be the most effective in reducing malaria transmission.^26^ Our previous study also provides some evidence that an intervention with azithromycin during the low-transmission season may be beneficial.^11^ It may be the case that single mass treatments during the low-transmission season provide protection, but repeated treatments would be required to reduce malaria risk during the high-transmission season. This study also did not include confirmatory antibiotic resistance testing, so it was not possible to examine the potential relationship between antibiotic resistance after 3 years of mass treatment and malaria parasitemia. Finally, this study only compared the effects of mass azithromycin on children receiving either annual or biannual treatment. A comparison of the effects of multiple rounds of mass azithromycin to no treatment could provide a more complete examination of the impact of mass azithromycin.

After 3 years of multiple distributions of mass azithromycin, we were unable to show a significant reduction in community-level prevalence of malaria parasitemia during the high-transmission season in communities randomized to biannual versus annual treatment. In this setting, additional mass azithromycin distributions during the low-transmission seasons did not reduce community-level prevalence of malaria parasitemia or parasite density. Mass azithromycin may have a modest impact on malaria prevalence during the low-transmission season, but there may be little protection from reinfection after a single treatment during the high-transmission season. Additional studies could examine malaria risk over time to better compare the impacts of different azithromycin distributions on malaria prevalence during both the low- and high-transmission seasons.
